# Structural insights into the inactivation of CRISPR-Cas systems by diverse anti-CRISPR proteins

**DOI:** 10.1186/s12915-018-0504-9

**Published:** 2018-03-19

**Authors:** Yuwei Zhu, Fan Zhang, Zhiwei Huang

**Affiliations:** HIT Center for Life Sciences, School of Life Science and Technology, Harbin Institute of Technology, Harbin, China

**Keywords:** CRISPR-Cas system, Anti-CRISPR, Adaptive immune, Viral infection, Structure

## Abstract

A molecular arms race is progressively being unveiled between prokaryotes and viruses. Prokaryotes utilize CRISPR-mediated adaptive immune systems to kill the invading phages and mobile genetic elements, and in turn, the viruses evolve diverse anti-CRISPR proteins to fight back. The structures of several anti-CRISPR proteins have now been reported, and here we discuss their structural features, with a particular emphasis on topology, to discover their similarities and differences. We summarize the CRISPR-Cas inhibition mechanisms of these anti-CRISPR proteins in their structural context. Considering anti-CRISPRs in this way will provide important clues for studying their origin and evolution.

## A microbial arms race

Prokaryotes and viruses have been engaged in an evolutionary “arms race” for billions of years. Prokaryotes employ CRISPR (clustered regularly interspaced short palindromic repeats)-Cas adaptive immune systems to protect against viral infection [1–6]. The CRISPR-Cas systems have been identified in about 50% of bacteria and 90% of archaea. A typical CRISPR locus is composed of an array of short direct repeats and interspersed spacer sequences (short DNA sequences from invading viruses), which is flanked by diverse *cas* genes. Based on the most recent phylogenetic classification, the CRISPR-Cas systems can be divided into two groups, which are further subdivided into six types and 19 subtypes [7–9]. Class I CRISPR-Cas systems, consisting of types I, III, and IV, are composed of a multi-subunit ribonucleoprotein complex named Cascade [10]. Class II CRISPR-Cas systems, consisting of types II, V, and VI, contain only a single nuclease protein [11–14]. The CRISPR-Cas immunity pathway is initiated through spacer acquisition, which integrates foreign DNA into the host CRISPR array locus [15–18]. Then, the CRISPR array locus is transcribed into a pre-crRNA (pre-CRISPR RNA). The pre-crRNA is further processed into mature crRNAs before being incorporated into the CRISPR-Cas interference modules [19]. Finally, the CRISPR-Cas effectors recognize and cleave the target foreign nucleic acids under the guidance of crRNA [20].

In turn, phages and mobile genetic elements encode a type of protein to destroy the highly prevalent CRISPR-Cas adaptive immune systems of prokaryotes, termed anti-CRISPR (Acr) [21–25]. Up to now, a total of 22 distinct families of anti-CRISPR genes have been reported. The proteins encoded by these genes are entirely distinct, with low sequence similarity. Based on the classification of target CRISPR-Cas immunity systems, these proteins are divided into two classes, Class I anti-CRISPRs and Class II anti-CRISPRs. To gain insights into the anti-CRISPR mechanisms, structural biologists have made great efforts to solve the structures of these anti-CRISPR proteins alone or in complex with the target CRISPR-Cas effectors. In all, the structures of seven anti-CRISPR proteins have been reported, including AcrF1, AcrF2, AcrF3, AcrF10, AcrIIA1, AcrIIA4, and AcrIIC1. However, a comprehensive and systematic structural analysis of these anti-CRISPR proteins is still lacking. In this review, we provide a snapshot of this ongoing molecular arms race, and aim to understand the inhibition mechanisms of these anti-CRISPR proteins from a structural perspective.

## A snapshot of the type I anti-CRISPRs

During the co-evolution of prokaryotes and viruses, prokaryotic CRISPR-Cas systems have evolved to select short sequences (protospacers) of the invading DNA and integrate them as spacer sequences into CRISPR loci to provide sequence-specific immunity [5, 26]. In this situation, it’s hard for viruses to escape the CRISPR-Cas adaptive immune systems [27–29]. Thus, anti-CRISPR genes found in bacteriophages may represent a widespread mechanism for phages to defeat the highly prevalent CRISPR-Cas adaptive immune systems. The type I CRISPR-Cas system can be divided into seven subtypes, I-A to I-F and I-U. In 2013, research characterizing the prophage-mediated phenotypes in the human pathogen *Pseudomonas aeruginosa* led to the identification of five phage-encoded anti-CRISPR genes, named *acrF1–5* [30]. These anti-CRISPR genes are specific to the type I-F CRISPR-Cas system of *P. aeruginosa*. A subsequent study discovered another four anti-CRISPR genes located at the same phage operons, which mediated inhibition of the type I-E CRISPR-Cas system of *P. aeruginosa* [31]. These genes are known as *acrE1–4*. In 2016, April Pawluk and colleagues developed a bioinformatics approach that enabled them to identify another five anti-CRISPR genes (*acrF6–10*) in diverse bacterial species, targeting the type I-F CRISPR-Cas system [32]. It is worth noting that one anti-CRISPR gene, *acrF6*, possesses a dual specificity and inhibits both type I-E and type I-F CRISPR-Cas systems.

**Fig. 1:**
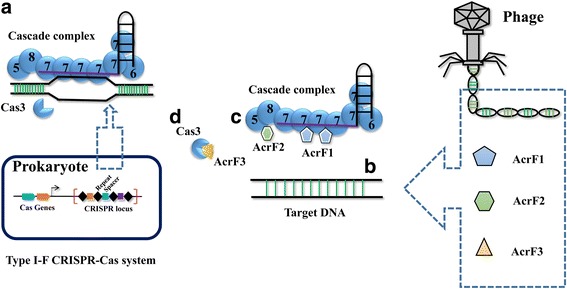
A cartoon depicting the architecture of the type I-F CRISPR-Cas system and inhibition mechanisms of three type I-F anti-CRISPRs. **a** The type I-F Csy complex is a 350-kDa crRNA-guided surveillance complex composed of a 60-nucleotide crRNA and nine Cas proteins, which recruits a nuclease-helicase protein Cas3 for target degradation. **b** AcrF1 interacts with Cas7f, sterically hindering target DNA access to the crRNA guide. **c** AcrF2 interacts with Cas8f and Cas7f, resembling a DNA duplex and sterically hindering target dsDNA access to the binding pocket. **d** AcrF3 forms a homodimer, interacting with Cas3 and impeding the recruitment of Cas3 to Cascade

These anti-CRISPR genes encode a set of small (~ 50–150 amino acids) proteins, lacking sequence similarity (Table 1). Bondy-Denomy and colleagues expressed four of these anti-CRISPR proteins (AcrF1–4) in vitro and performed biochemical experiments to investigate the mechanisms by which they inhibit the type I-F Csy complex [33]. Type I-F Csy complex is a 350 kDa crRNA-guided surveillance complex composed of a 60-nucleotide crRNA and nine Cas proteins (one Cas8f, one Cas5f, one Cas6f, and six Cas7f), which recruits a nuclease-helicase protein Cas3 for target degradation (Fig. 1a) [34, 35]. The biochemical results show that AcrF1 and AcrF2 interact directly with the Csy complex and block its binding with the DNA target (Fig. 1b, c). AcrF3 binds directly to the Cas3 nuclease and hinders its recruitment to the DNA-bound Csy complex (Fig. 1d). AcrF4 also interacts with the Csy complex, yet the inhibition mechanism is still elusive. To deepen our understanding of the interaction architectures of anti-CRISPR proteins with the Csy complex and elucidate the precise inhibition mechanisms, we need to be able to visualize the structures of anti-CRISPRs bound to their target.

**Table 1:** Anti-CRISPR proteins and their mechanisms of action

Anti-CRISPR (source)	Size (amino acids)	CRISPR inhibited	Inhibition mechanism	Structure (PDB code)	Citation
AcrE1 (*P. aeruginosa*)	100	Type I-E	–	–	[31]
AcrE2 (*P. aeruginosa*)	84	Type I-E	–	–	[31]
AcrE3 (*P. aeruginosa*)	68	Type I-E	–	–	[31]
AcrE4 (*P. aeruginosa*)	52	Type I-E	–	–	[31]
AcrF1 (*P. aeruginosa*)	78	Type I-F	Inhibits DNA binding	2LW5,5UZ9,6ANV,6B46	[30, 36–39]
AcrF2 (*P. aeruginosa*)	90	Type I-F	Partially overlaps with the binding site of dsDNA	5UZ9,6B47	[30, 36, 39]
AcrF3 (*P. aeruginosa*)	139	Type I-F	Blocks the entrance of the DNA binding tunnel; blocks new sequence acquisition	5GNF,5GQH,5B7I	[30, 40–42]
AcrF4 (*P. aeruginosa*)	100	Type I-F	–	–	[30]
AcrF5 (*P. aeruginosa*)	79	Type I-F	–	–	[30]
AcrF6 (*P. aeruginosa*)	100	Type I-E/F	–	–	[32]
AcrF7 (*P. aeruginosa*)	67	Type I-F	–	–	[32]
AcrF8 (*P. atrosepticum*)	92	Type I-F	–	–	[32]
AcrF9 (*V. parahaemolyticus*)	68	Type I-F	–	–	[32]
AcrF10 (*S. xiamenensis*)	97	Type I-F	DNA mimic, blocks DNA binding	6ANW,6B48	[32, 39]
AcrIIA1 (*L. monocytogenes*)	149	Type II-A	–	5Y6A	[51, 60]
AcrIIA2 (*L. monocytogenes*)	123	Type II-A	Inhibits DNA binding	–	[51]
AcrIIA3 (*L. monocytogenes*)	125	Type II-A	–	–	[51]
AcrIIA4 (*L. monocytogenes*)	87	Type II-A	PAM mimic, inhibits DNA binding; interacts with active site within the RuvC domain; hinders the conformation change of the HNH domain	5XBL,5VW1,5VZL	[51, 55–57]
AcrIIA5 (*S. thermophilus*)	140	Type II-A	–	–	[54]
AcrIIC1 (*N. meningitidis*)	85	Type II-C	Binds the HNH domain; shields the catalytic center	5VGB	[50, 59]
AcrIIC2 (*N. meningitidis*)	123	Type II-C	–	–	[50]
AcrIIC3 (*N. meningitidis*)	116	Type II-C	Induces Cas9 dimerization; inhibits DNA binding	–	[50, 59]

### Structure and inhibition mechanism of AcrF1

**Fig. 2:**
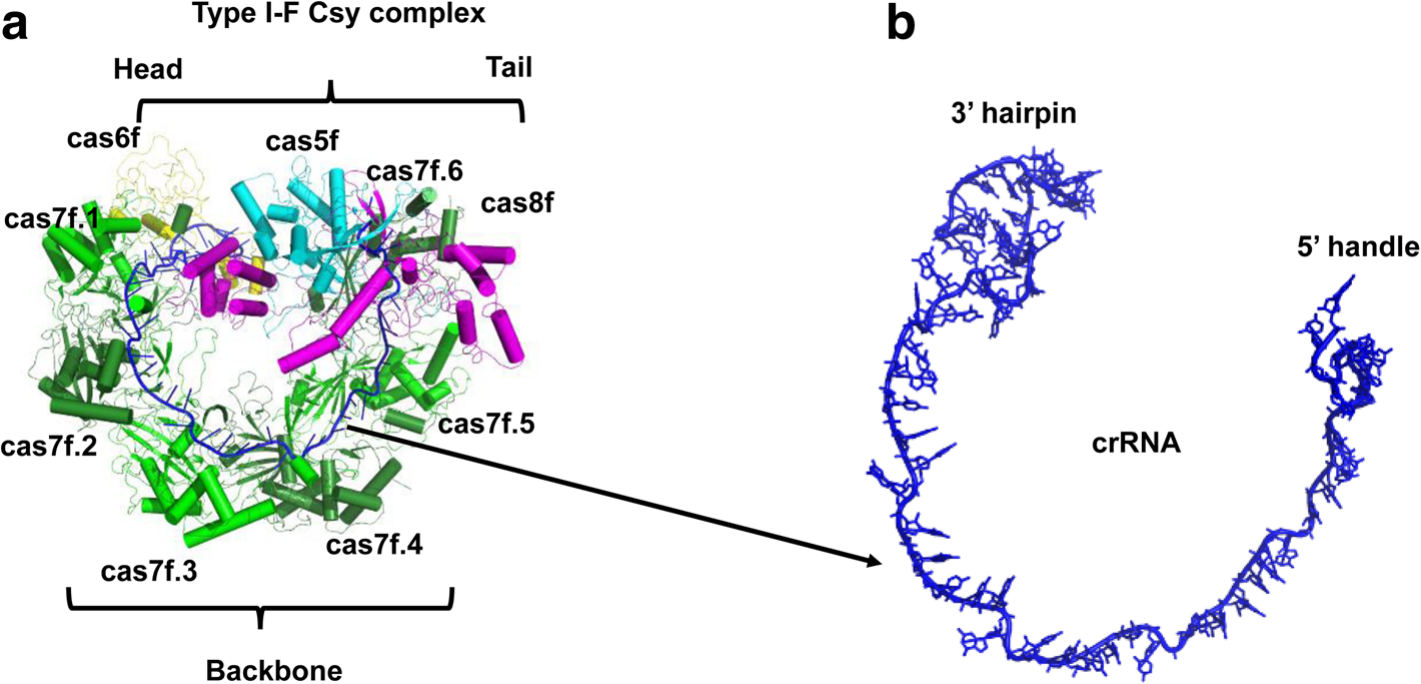
Cartoon view of the structure of the type I-F Csy complex. **a** Structure of the type I-F Csy complex. The cas5f, cas6f, cas7f, and cas8f subunits of the type I-F Csy complex are colored *cyan*, *yellow*, *green*, and *magenta*, respectively. The crRNA is colored blue. **b** An enlarged view of the structure of crRNA, which resembles a string

**Fig. 3:**
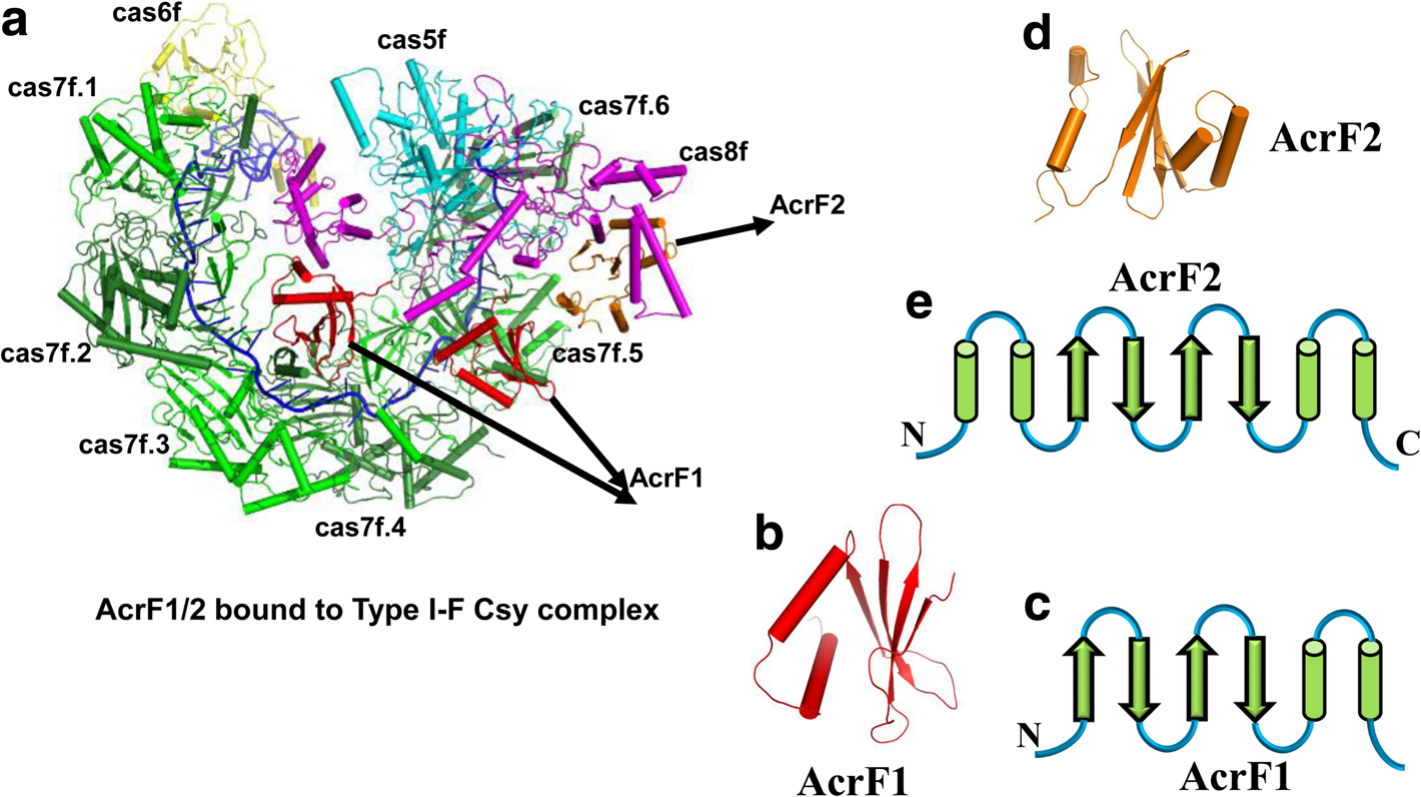
Cartoon view of the structure of the type I-F Csy complex bound to AcrF1/2. **a** Structure of the type I-F Csy complex bound to two anti-CRISPR proteins, AcrF1 and AcrF2. The cas5f, cas6f, cas7f, and cas8f subunits of the type I-F Csy complex are colored as Fig. 2a. AcrF1 and AcrF2 are colored *red* and *orange*, respectively. 3D structure (**b**) and topological view (**c**) of AcrF1. 3D structure (**d**) and topological view (**e**) of AcrF2. Helices and strands are shown as *green cylinders* and *arrows*, respectively

The cryo-EM structure of the type I-F Csy complex bound to two different Acr proteins, AcrF1 and AcrF2, was first determined by Chowdhury and colleagues at an average resolution of 3.4 Å [36]. The overall structure of type I-F Csy complex shows a nearly closed ring architecture, with the Cas6f head, Cas7f backbone, and Cas8f-Cas5f tail (Fig. 2a). The 60-nucleotide crRNA plays an essential structural role in complex assembly, resembling a string and tethering the protein subunits of this complex together (Fig. 2b). The AcrF1 protein encoded by gene 35 from *P. aeruginosa* phage JBD30 consists of 78 amino acids (Table 1). The overall structure of AcrF1 adopts a very simple fold, composed of four anti-parallel β-strands and two anti-parallel α-helices (β1↑-β2↓-β3↑-β4↓-α1-α2) (Fig. 3a–c). These four anti-parallel β-strands constitute a β-sheet packing against the C-terminal two anti-parallel α-helices to form a hydrophobic core (Fig. 3b). The structure of AcrF1 bound to the type I-F Csy complex matches well with that of AcrF1 determined by the nuclear magnetic resonance (NMR) method [37]. In this complex structure, two copies of AcrF1 are observed to interact with the Cas7f backbone (Fig. 3a). One molecule binds to the interface formed by Cas7f.3 and Cas7f.4, and the other recognizes the interface formed by Cas7f.5 and Cas7f.6 (Fig. 3a). From the combination of biochemical and structural information, we know that two AcrF1 proteins sit on top of the Cas7f.4 and Cas7f.6 thumbs, sterically hindering the access of the crRNA guide to target DNA. In addition, AcrF1 proteins interact with the basic residues on the Cas7f molecules that are crucial for target DNA binding.

Shortly afterwards, Gao’s group reported another AcrF1-alone binding mode [38]. Significantly different from the AcrF1 and AcrF2 co-binding mode, the AcrF1-alone binding mode consists of three copies of AcrF1. Two molecules bind to the equivalent interface formed by Cas7f.2-Cas7f.3 and Cas7f.4-Cas7f.5. The third AcrF1 interacts with Cas7f.6, which is in close proximity to the Cas8f-Cas5f tail, thus occupying the AcrF2 binding site. These two binding modes are consistent with previous studies that either AcrF1 or AcrF2 is sufficient to inhibit the Csy complex-mediated CRISPR-Cas immunity [30, 33]. However, how the AcrF1 molecules inhibit the type I-F Csy complex individually and how many copies of AcrF1 are sufficient to block the target DNA binding are unknown. A Coomassie staining result reported by Bondy-Denomy et al. showed the stoichiometry of AcrF1 to be 2.6 proteins per Csy complex [33]. Recently, Guo et al. also determined the cryo-EM structure of the Csy^crRNA^-AcrF1 complex [39]. The structure shows that AcrF1 binds at the same position and with the same stoichiometry as reported by Chowdhury et al. However, due to the insufficient EM density, tail subunits Cas5f and Cas8f could not be traced. Thus, it is still unclear whether the third AcrF1 binding site (Cas7f.6) is important.

### Structure and inhibition mechanism of AcrF2

AcrF2 protein encoded by gene 30 from *P. aeruginosa* phage D3112 is a small acidic protein consisting of 90 amino acids (Table 1). The structure of AcrF2 bound to the type I-F Csy complex was determined by single-particle cryo-EM (Fig. 3a) [36, 39]. The overall structure of AcrF2 displays a sandwich fold, comprising four anti-parallel β-strands flanked by two α-helices at both sides (α1-α2-β1↑-β2↓-β3↑-β4↓-α3-α4; Fig. 3d, e). The topological structure of AcrF2 is very similar to that of AcrF1, with the addition of two α-helices at the N-terminus.

In the cryo-EM structure, AcrF2 binds between the thumb of Cas7f.6 and the hook of Cas8f (Fig. 3a) [36, 39]. A noticeable structural feature of AcrF2 is that lots of acidic residues on the surface exhibit a pseudo-helical distribution, such that the structure of AcrF2 resembles a DNA duplex. Coincidentally, surrounding the acidic residues at the interaction interface are numerous positively charged residues on either the thumb of Cas7f or the N-terminal hook of Cas8f, which are critical for DNA binding. Compared with the recently reported Csy^crRNA^–dsDNA complex structure, we know that the binding site of AcrF2 partially overlaps with that of the dsDNA, and the binding of AcrF2 pushes the hook domain of Cas8f away from the dsDNA binding pocket [39]. Collectively, we conclude that AcrF2 sterically hinders the access of target dsDNA to the binding pocket of type I-F Csy complex. In addition, AcrF2 interacts with the surrounding basic residues of type I-F Csy complex vital for target DNA binding and keeps the DNA binding domain far away.

### Structure and inhibition mechanism of AcrF3

**Fig. 4:**
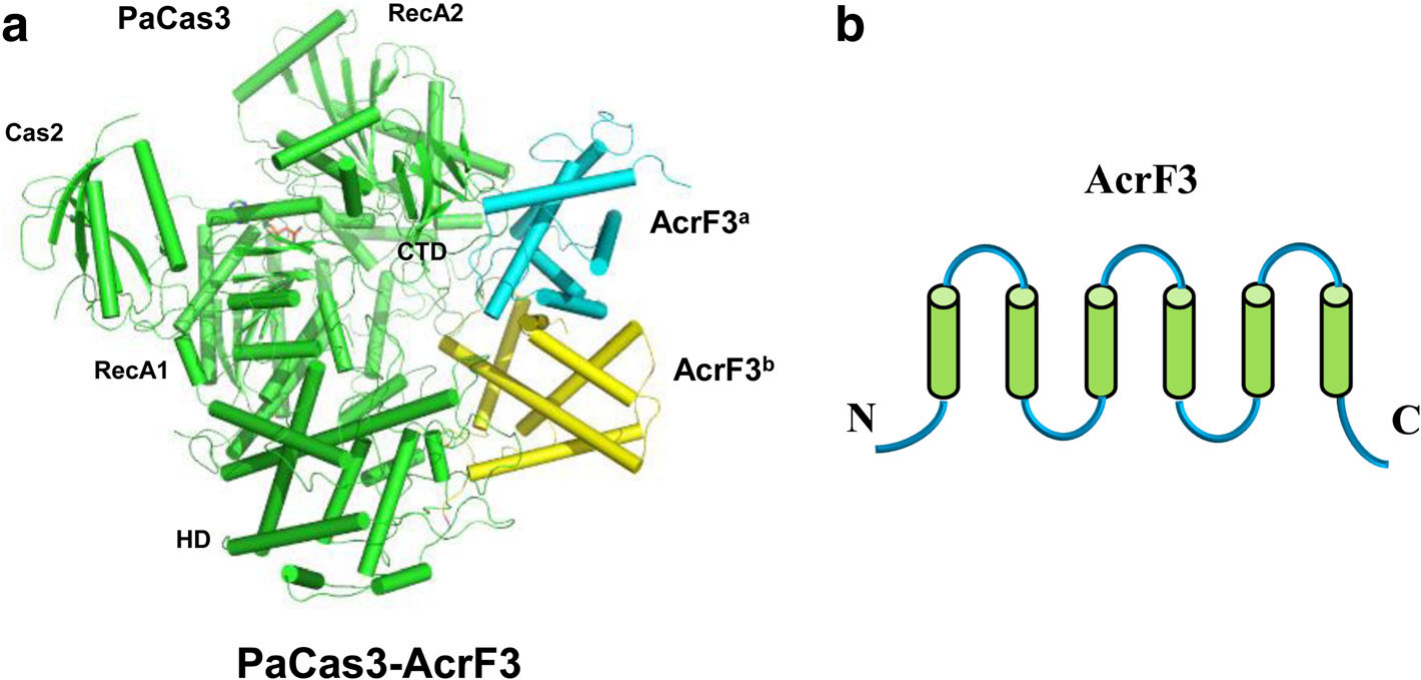
Cartoon view of the structure of PaCas3 in complex with AcrF3. **a** Structure of the PaCas3–AcrF3 complex. PaCas3 is colored *green*. The AcrF3 dimer is colored *cyan* and *yellow*, respectively. The Cas2, RecA1/2, HD, and CTD domains of PaCas3 are labeled. **b** Topological view of AcrF3

The AcrF3 protein, encoded by gene 35 from *P. aeruginosa* phage JBD, is larger than either AcrF1 or AcrF2, consisting of 139 amino acids (Table 1). In 2016, Zhu’s group determined the crystal structure of AcrF3 in complex with PaCas3 at a resolution of 2.6 Å [40, 41]. Subsequently, Wang and colleagues reported the crystal structure of AcrF3 at a resolution of 1.5 Å, and solved a 4.2-Å cryo-EM structure of PaCas3 (residue 106–1076)–AcrF3 complex using the cryo-EM single particle method [42]. The overall structure of AcrF3 is composed of six α-helices (Fig. 4a, b). Consistent with the gel-filtration chromatography results, the structure of AcrF3 exhibits a dimeric architecture [42]. The AcrF3 dimer sits on a groove enclosed by the HD domain, RecA1, RecA2, and the CTD of PaCas3 (Fig. 4a) [40]. PaCas3 and AcrF3 form a compact complex via numerous hydrogen bonds and extensive hydrophobic interactions. In the type I CRISPR-Cas adaptive immune systems, Cascade binds invading DNA and generates an R loop under the guidance of crRNA [43]. Then, the Cas3 helicase-nuclease is recruited to the R loop region, where it unwinds and degrades target DNAs [44, 45].

Through comparing the structures of the PaCas3-AcrF3 and TfCas3-DNA, Zhu’s group found that the 5′ end of a single-stranded DNA molecule is bound in a previously mentioned groove [40, 46]. However, the entrance of this groove is completely blocked by the AcrF3 dimer, as observed in the structure of the PaCas3–AcrF3 complex, indicating that AcrF3 inhibits the activity of PaCas3 through sterically hindering the access of the substrate DNA to PaCas3. Furthermore, Wang and colleagues provide a new insight; that inhibiting degradation of target DNAs, through the AcrF3 dimer hindering the recruitment of Cas3 to the Csy–dsDNA complex, also hampers the generation of the precursor protospacer DNA, thus impeding both crRNA interference and spacer acquisition [42, 47, 48]. Recently, Rollins et al. revealed that AcrF3 does not prevent Cas3 nuclease activity directly. In addition, AcrF3 binds directly to the Cas1–2/3 complex, which may explain how AcrF3 blocks new sequence acquisition [49].

### Structure and inhibition mechanism of AcrF10

**Fig. 5:**
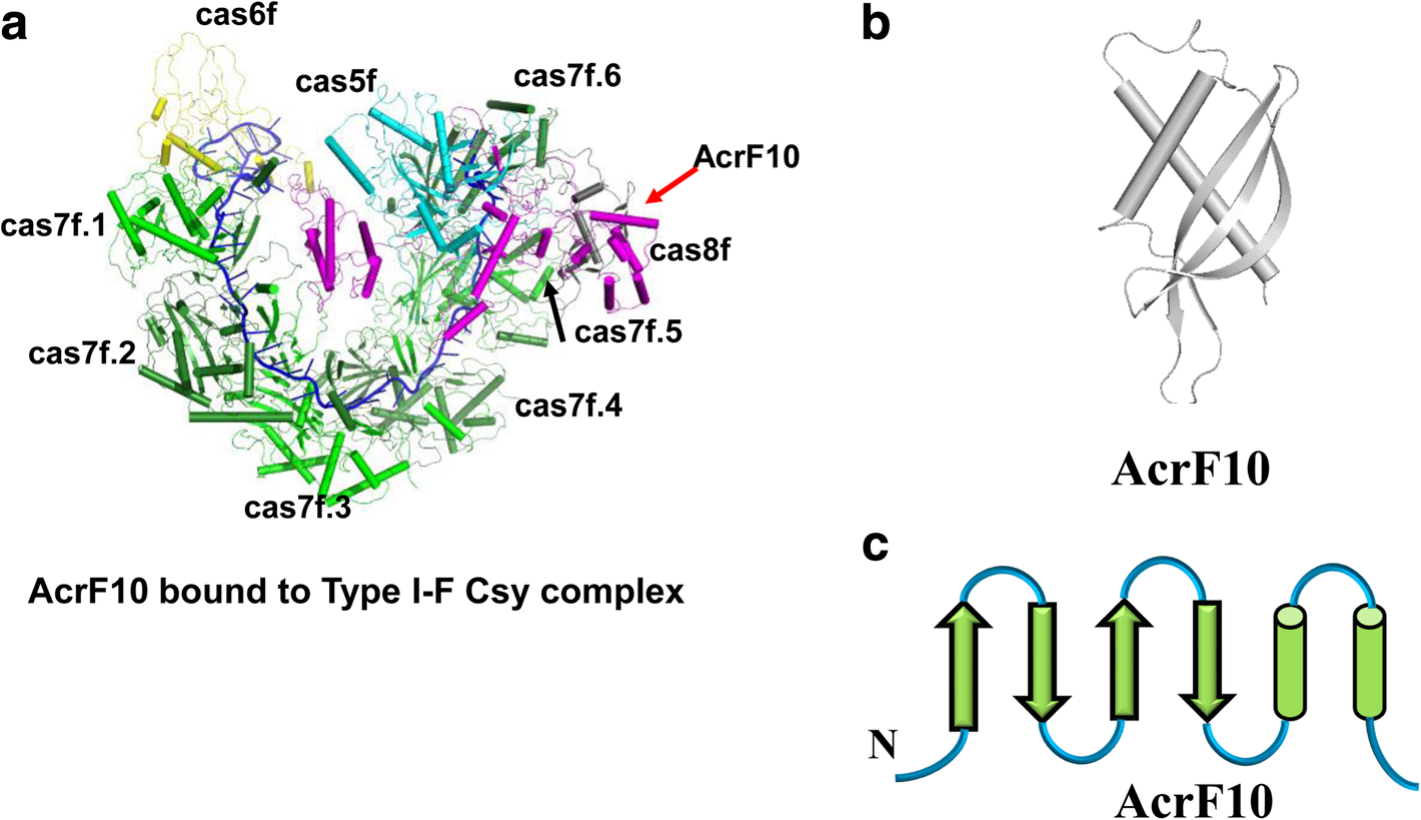
Cartoon view of the structure of the type I-F Csy complex bound to AcrF10. **a** Structure of the type I-F Csy complex bound to AcrF10. The cas5f, cas6f, cas7f, and cas8f subunits of the type I-F Csy complex are colored as Fig. 2a. AcrF10 is colored *black*. 3D structure (**b**) and topological view (**c**) of AcrF10

AcrF10 from a prophage of *Shewanella xiamenensis* is also a small acidic protein, consisting of 97 amino acids (Table 1). Size-exclusion chromatography (SEC) results suggest that AcrF10 could form a stable complex with Cas5f-Cas8f in vitro [39]. The structure of AcrF10 was determined by X-ray diffraction [39]. To explore the inhibition mechanism of AcrF10, an atomic structure of the Csy^crRNA^–AcrF10 complex was determined by single-particle cryo-EM at a resolution of 3.6 Å [39]. The architecture of AcrF10 adopts a simple α/β fold, comprising a four-stranded β-sheet with three α-helices at one side (Fig. 5a–c).

In the cryo-EM structure, AcrF10 is situated in a groove formed by Cas7f.6 and the Cas8f hook (Fig. 5a). For the structure of the Csy^crRNA^–dsDNA complex, the equivalent groove is observed just for accommodating the target double-stranded DNA [39]. In addition, AcrF10 interacts with the surrounding basic residues vital for target DNA binding. Similar to DNA binding, AcrF10 binding causes a conformational change of the Cas8f hook domain, which moves toward Cas7f.6. Guo et al. conclude that AcrF10 acts as a DNA mimic, while they dispute the claim that AcrF2 is one [39].

## A snapshot of the type II anti-CRISPRs

Employing the same bioinformatic method that successfully identified type I anti-CRISPR genes, Pawluk and colleagues discovered the *acrIIC1* gene from *Brackiella oedipodis* [50]. The inhibition activity of *acrIIC1* genes from *B. oedipodis* and its homolog, bearing 29% sequence identity from *Neisseria meningitides*, was successfully identified in the *N. meningitidis* strain 8013, which harbors the best-established type II CRISPR-Cas system [50]. The type II CRISPR-Cas system of *N. meningitidis* was inhibited by these two anti-CRISPR genes, named *acrIIC1*_Boe_ and *acrIIC1*_Nme_. Subsequently, another two genes (*acrIIC2*_Nme_ and *acrIIC3*_Nme_) with robust anti-CRISPR activity were also identified in *N. meningitidis* [50]. These three type II-C anti-CRISPR proteins (AcrIIC1Nme, AcrIIC2Nme, and AcrIIC3Nme) interact directly with sgRNA-loaded NmeCas9 and inhibit the NmeCas9 enzymatic activity both in vitro and in vivo [50].

Almost simultaneously, Bondy-Denomy’s group reported an innovative bioinformatics approach to screening for CRISPR-Cas inhibitor genes using “self-targeting” as a genomic marker [51]. “Self-targeting” is a common phenomenon observed in the intracellular food-borne pathogen *Listeria monocytogenes* [52]. In many *L. monocytogenes* isolates, the space–protospacer pairs are able to coexist in the presence of type II-A CRISPR-Cas systems, indicating that these genomes may encode anti-CRISPRs [53]. *L. monocytogenes* strain J0161 contains an apparently self-targeted sequence; thus, the prophage ΦJ0161a is considered as a source of inhibitor genes [51]. Comparing the genome of prophage ΦJ0161a with that of its closely related prophage Φ10403s, two anti-CRISPR genes *acrIIA1* and *acrIIA2* were identified [51]. Using BLAST searches with the genomic position analogous to that of these two anti-CRISPR genes in related *L. monocytogenes* prophages leads to the identification of another two type II-A CRISPR-Cas9 inhibitors (*acrIIA3* and *acrIIA4)* [51]. It is worth noting that AcrIIA2 and AcrIIA4 possess broad specificity, inhibiting the activity of both the LmoCas9 and SpyCas9, which share 53% sequence identity.

Recently, another anti-CRISPR gene, *acrIIA5*, was discovered in a *Streptococcus thermophilus* virulent phage [54]. Gene *acrIIA5* encodes a 140-amino-acid protein, inhibiting the activity of type II-A Cas9 from both *S. pyogenes* and *S. thermophilus*. AcrIIA5 is predicted to be structurally distinct from other characterized anti-CRISPR proteins, containing a putative coiled-coil motif that might possess the nucleic acid binding ability.

### Structure and inhibition mechanism of AcrIIA4

To understand the mechanism of AcrIIA2- or AcrIIA4-mediated Cas9 inhibition and better utilize these “off-switch” tools, our group determined the crystal structure of AcrIIA4 in complex with SpyCas9 and a single-guide RNA (sgRNA) at a resolution of 3.0 Å [55]. Subsequently, Yang et al. and Shin et al. reported the same AcrIIA4–SpyCas9–sgRNA complex structure using X-ray diffraction and cryo-EM, respectively [56, 57].

**Fig. 6.**
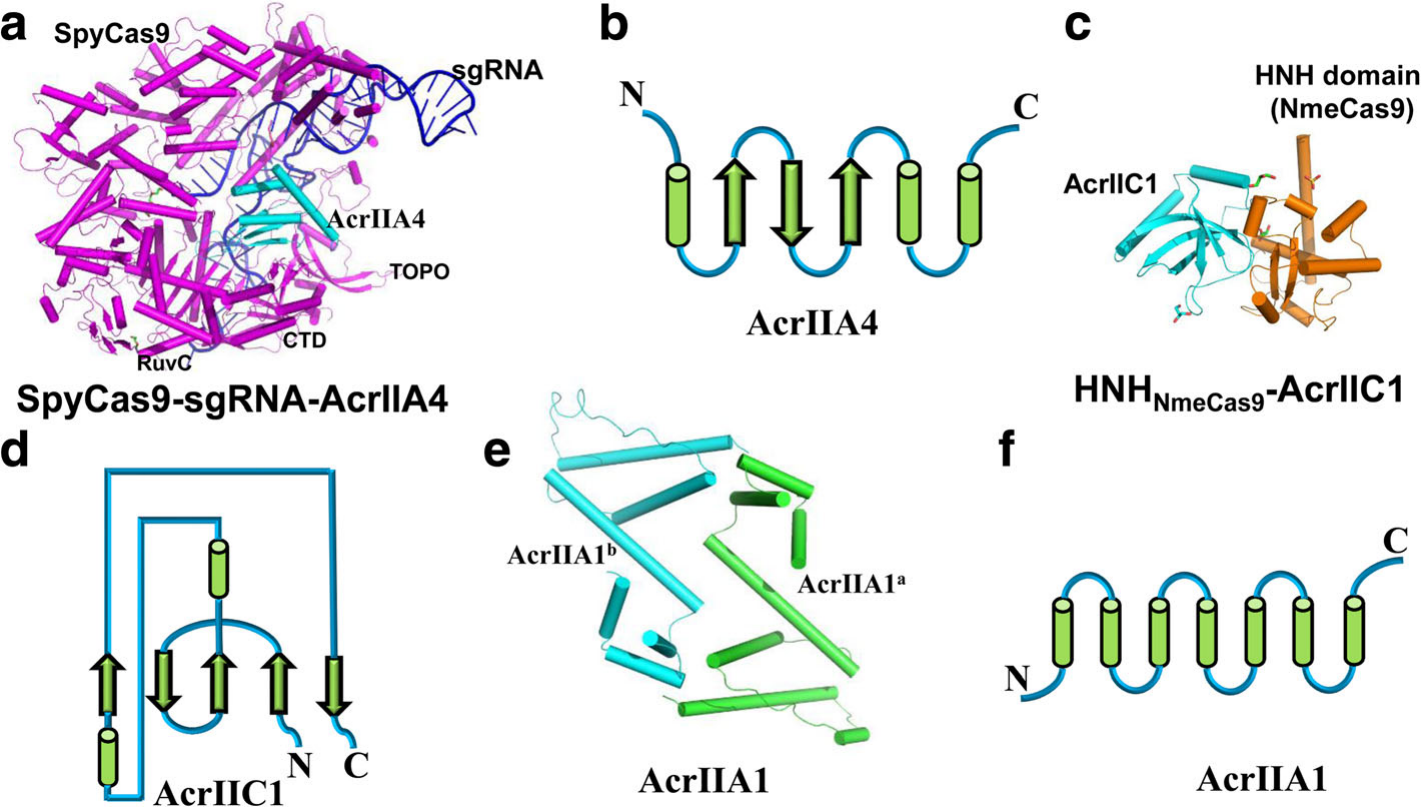
Cartoon and topological views of the structures of type II anti-CRISPR proteins. **a** Structure of the AcrIIA4–SpyCas9–sgRNA complex. SpyCas9 and AcrIIA4 are colored *magenta* and *cyan*, respectively. The crRNA is colored *blue*. The RuvC, CTD, and TOPO domains of SpyCas9 are labeled. **b** Topological view of AcrIIA4. **c** Structure of the AcrIIC1–HNH_NmeCas9_ domain complex. AcrIIC1 and the HNH_NmeCas9_ domain are colored *cyan* and *orange*, respectively. **d** Topological view of AcrIIC1. **e** Structure of the AcrIIA1 dimer. The AcrIIA1 dimer is colored *cyan* and *green*. **f** Topological view of AcrIIA1

AcrIIA4 from an *L. monocytogenes* prophage consists of 87 amino acids (Table 1). The electrostatic potential distribution indicates that AcrIIA4 is also an acidic protein, similar to that of AcrF2 and AcrF10. The overall structure of AcrIIA4 displays a “triangle” fold, composed of three anti-parallel β-strands and three α-helices (α1-β1↑-β2↓-β3↑-α2-α3; Fig. 6a, b) [55]. These three anti-parallel β-strands constitute a β-sheet, with three α-helices at one side. The topological structure of AcrIIA4 is similar to that of AcrF1, with the first α-helix replaced with a β-strand. In the structure of AcrIIA4–SpyCas9–sgRNA, AcrIIA4 occupies the PAM-interacting site, interacting with the TOPO, CTD, and RuvC domains of spyCas9 (Fig. 6a) [55]. Structural comparison of AcrIIA4–SpyCas9–sgRNA and SpyCas9–sgRNA–dsDNA suggests that AcrIIA4 inhibits the nuclease activity of SpyCas9 through multiple mechanisms [55, 58]: (1) sterically blocking the PAM-binding site and interacting with the surrounding basic residues vital for PAM recognition; (2) interacting with the residues in the phosphate lock loop and occupying the + 1 phosphate group of target dsDNA to inhibit target dsDNA unwinding; (3) interacting with the active site within the RuvC domain and blocking the entrance of the non-target strand into the RuvC active site; (4) hampering the conformation change of HNH (two pairs of conserved histidines and one asparagine) domain—which cleaves the DNA strand complementary to the RNA guide—from the inactive to the active state.

### Structure and inhibition mechanism of AcrIIC1

AcrIIC1 is a broad-spectrum type II-C anti-CRISPR, inhibiting divergent Cas9 orthologs (NmeCas9, CjeCas9, and GeoCas9) both in vitro and in vivo [50]. Biochemical results indicate that AcrIIC1 interacts directly with the HNH domain and blocks DNA cleavage via trapping Cas9 on its DNA target in a catalytically inactivated state [59]. To elaborate the detailed inhibition mechanism, the Doudna group determined the crystal structure of AcrIIC1 in complex with the HNH domain of NmeCas9 at a resolution of 1.5 Å [59]. AcrIIC1 from an *N. meningitidis* prophage consists of 85 amino acids (Table 1). The overall structure of AcrIIC1 adopts a novel fold, comprising five β-strands and two α-helices (β1↑-β2↓-β3↑-α1-α2-β4↑-β5↓; Fig. 6c, d). These five β-strands constitute a β-barrel, with two α-helices at the C-terminal side (Fig. 6c, d).

As with PaCas3 and AcrF3, a stable interaction between AcrIIC1 and the HNH domain of NmeCas9 is supported by numerous hydrogen bonds and extensive hydrophobic interactions observed in the interface. Sequence alignment of HNH domain homologues from multiple species indicates that residues at the binding interface are highly conserved between *N. meningitidis*, *C. jejuni*, and *G. stearothermophilus*, but not *S. pyogenes* [59]. This is perfectly consistent with the DNA cleavage results using various types of II-C Cas9 orthologs [59]. In the complex structure of AcrIIC1–NmeCas9 HNH domain, residues of AcrIIC1 form hydrogen bonds with the highly conserved catalytic residues of the HNH domain, thus shielding the HNH catalytic domain and inhibiting the DNA cleavage activity [59]. It is a novel inhibition strategy compared with that adopted by the type II-A anti-CRISPR, AcrIIA4. On the other hand, AcrIIC1 may exist as a gene regulator, due to the fact that AcrIIC1-targeted Cas9 orthologs still retain RNA-programmed DNA binding ability.

### Structure and inhibition mechanism of AcrIIA1

AcrIIA1 from prophage of *L. monocytogenes* is a prevalent type II-A anti-CRISPR, consisting of 149 amino acids (Table 1). Recently, the structure of AcrIIA1 was determined by X-ray diffraction at a resolution of 2.0 Å [60]. The structure reveals that two AcrIIA1 molecules form a homodimer, each of which displays an all-helical two-domain architecture (Fig. 6e, f). The N-terminal domain shows structural similarity to the HTH domain of many transcription factors. The C-terminal domain exhibits a structural feature with unknown function. When overexpressed in *Escherichia coli*, AcrIIA1 was found to associate with heterogeneous RNA [60]. Ka and colleagues [60] speculate that AcrIIA1 may function via RNA recognition for inactivation of CRISPR-Cas immunity systems. However, the precise inhibition mechanism is still elusive.

## Similarities and differences in structural architectures and inhibition strategies

As described above, up to now, seven structures of anti-CRISPR proteins have been determined, consisting of AcrF1, AcrF2, and AcrF10 that target the *P. aeruginosa* type I-F Csy complex, AcrF3 that targets the *P. aeruginosa* type I-F helicase-nuclease Cas3, AcrIIA4 that targets *S. pyogenes* type II-A Cas9, AcrIIC1 that targets type II-C Cas9, and AcrIIA1 [36, 38–40, 42, 55–57, 59]. These anti-CRISPR proteins share very low sequence identity and display divergent architectures. However, structural similarities of these anti-CRISPR proteins can be found. For example, AcrF1, AcrF2, AcrF10, and AcrIIA4 all adopt the α/β fold, comprising a variant β-sheet flanked by α-helices on one side or both side. Moreover, these proteins adopt an analogous inhibition mechanism of blocking target DNA binding. The difference between them is that AcrF1 blocks the single-stranded DNA hybridizing with crRNA, while AcrF2, AcrF10, and AcrIIA4 prevent PAM duplex access to its binding site. More importantly, we found that the binding interfaces of these anti-CRISPRs are mainly located at the β-strand region (or loops connecting β-strands). The α-helices seem to function to enclose the hydrophobic hole to stabilize the protein structure.

AcrIIC1 also displays an α/β fold, with five β-strands constituting a β-barrel instead of a β-sheet [59]. Unique among these anti-CRISPR proteins, AcrF3 is composed entirely of α-helices [40, 42]. In addition, the active state of AcrF3 exhibits a dimeric architecture, distinguishing it from the monomeric anti-CRISPRs mentioned above. Similarly, these two proteins both adopt an inhibition strategy of targeting nucleases or nuclease domains. The difference is that AcrIIC1 interacts directly with the catalytic residues of the nuclease domain, thus shielding the catalytic activity [59]. However, AcrF3 forms a dimer, covering the target DNA recognition groove to inhibit the nuclease activity. Beyond simple inhibition of interference, these two anti-CRISPR proteins possess the ability to convert the CRISPR-Cas system into a transcriptional repressor. AcrIIA1 is also composed entirely of α-helices, but the actual inhibition mechanism is still unclear.

In addition, although structural information is still lacking, biochemical results indicate that AcrIIA2 adopts a similar inhibition mechanism to AcrIIA4, interacting directly with sgRNA-bound SpyCas9, and blocking target dsDNA binding [55, 56]. In contrast, AcrIIC3 employs a novel inhibition strategy. The electron microscopy result shows that AcrIIC3 could induce dimerization of the sgRNA-bound NmeCas9 and reduce the binding affinity of NmeCas9 for target DNA [59].

## CRISPR-Cas systems and anti-CRISPRs evolve dependently on each other

Balance is a widespread natural principle in the evolution of species [61–63]. The phage–bacterial arms race is a typical case to study this behavior. In order to survive, bacteria exploit multiple types of CRISPR-Cas immune systems to fight against phages, and phages evolve diverse anti-CRISPRs to invade. Although lots of anti-CRISPRs have been successfully identified, there is a long way for us to go to unveil the details of this evolutionary war. Quite a few issues are waiting to be addressed. Firstly, what are the origins of anti-CRISPR genes? Anti-CRISPR genes are considered as accessory genes of phage [24]. These genes differ significantly from the core genes, which are vital for lytic or lysogenic replication under all conditions [64, 65]. An obvious characteristic of these accessory genes is that they render phages better able to adapt to a specific host. Given the close relationship between phages and hosts, we speculate that scientists could find some clues from hosts for the origins of anti-CRISPR genes.

Secondly, why are anti-CRISPR genes so diverse? We speculate that it may be caused by different origins of anti-CRISPR genes, or distinct evolutionary routes to adapt to the diverse CRISPR-Cas modules. Multiple evolutionary pathways may be adopted by phages to adapt to their respective host. We support the viewpoint that the prokaryotic CRISPR-Cas immune systems provide strong selection for the evolution of sophisticated virus-encoded anti-CRISPR mechanisms [29]. Moreover, we find that lots of anti-CRISPR genes share a common genomic neighborhood. For example, in *P. aeruginosa* phages, an *acrE* gene often borders an *acrF* gene in their anti-CRISPR loci [30, 31]. Similarly, *acrIIC2* and *acrIIC3* anti-CRISPR genes exist alongside each other in *N. meningitides* [50]. Moreover, *acrIIA2* and *acrIIA3* are nearly always found with *acrIIA1* in *L. monocytogenes* [51]. We propose that the phages carrying these genes may cycle through hosts with different CRISPR-Cas systems, or these anti-CRISPRs require potential functional complementation.

Thirdly, how to build the enigmatic anti-CRISPR arsenal. CRISPR-Cas systems can be divided into two classes based on the subunit component. Each class contains at least three types and multiple subtypes. Besides the prevailing Cas9, some other type II CRISPR-Cas effector proteins have been identified, such as type V-A Cpf1 (Cas12a), V-B C2c1 (Cas12b), V-C C2c3, VI-A C2c2 (Cas13a), and VI-B Cas13b [11, 66–70]. However, anti-CRISPRs targeting these effector proteins have still not been found. More bioinformatic tools should be developed to build the complete anti-CRISPR arsenal.

Fourthly, what are the inhibition mechanisms of all anti-CRISPRs. It has been well established that the CRISPR-Cas immunity response functions in three stages: spacer acquisition, crRNA biogenesis, and target inference. In theory, anti-CRISPRs can inhibit any stage of this process to block the CRISPR-Cas immunity response. Actually, biochemically identified inhibition mechanisms of these anti-CRISPR genes mainly focus on the destruction of the third stage of the CRISPR-Cas immunity response. The mechanisms other anti-CRISPR genes use to accomplish this task deserve further study. For example, hijacking the Cas proteins to disrupt the assembling of type I CRISPR-Cas systems or encoding RNAs to mimic crRNA are both good choices.

Last but not the least, do anti-anti-CRISPRs exist? In nature there is a balance between species, and no one species can reproduce without limit. We believe that if anti-CRISPRs defeat CRISPR-Cas immune systems, anti-anti-CRISPRs would appear.

## Learning lessons from anti-CRISPR architecture

Anti-CRISPRs are a recent discovery utilized by phages to fight against CRISPR-Cas-acquired resistance in prokaryotes. A striking feature of anti-CRISPRs is that these proteins are diverse and lack sequence similarity. To probe the origin and evolution of these anti-CRISPRs, a comprehensive and careful analysis of anti-CRISPR proteins has been performed. Here we have reviewed the structural and functional similarities and differences between anti-CRISPRs. Especially, we propose that scientists should not be spectators of this bacteria–phage arms race. The human species lives through wars and suffers from diseases, the invasion of pathogenic bacteria and viruses, and natural disasters. We should make more effort to study this microbial evolutionary war and draw a lot of lessons from it. For example, through investigating the phage–bacteria interaction, we can better understand the molecular mechanism of the arms race between viruses and their hosts. To date, by utilizing the CRISPR-Cas adaptive immune system from bacteria, scientists have made great progress in developing effective gene editing tools, with CRISPR-Cas9 technologies now being the most commonly used and powerful genome engineering tools [71–77]. However, side effects resulting from alternative cleavage still occur [78–82]. The discovery of anti-CRISPRs provides a lamp, highlighting the way to regulate the genome editing activities of CRISPR-Cas9. By utilizing the anti-CRISPRs from phages, we hope to develop a braking system for gene editing, to make sure that therapies based on gene editing can be fully controlled.
